# Gender and leg-dominance differences in shoe properties and foot injuries in badminton: a cross-sectional survey

**DOI:** 10.1186/s13047-022-00531-w

**Published:** 2022-04-04

**Authors:** Siqin Shen, Wing-Kai Lam, Jin Teng, Sheng-Wei Jia, Julien S. Baker, Ukadike C. Ugbolue, Gusztáv Fekete, Yaodong Gu

**Affiliations:** 1 Faculty of Sports Science, Ningbo University, Ningbo, 315211 China; 2 Savaria Institute of Technology, Eötvös Loránd University, Szombathely, 9700 Hungary; 3 Sports Information and External Affairs Centre, Hong Kong Sports Institute, Sha Tin, Hong Kong; 4 Department of Sports Biomechanics, Beijing Sport University, Beijing, 100084 China; 5 Li Ning Sports Sciences Research Center, Li Ning (China) Sports Goods Limited Company, Beijing, 101111 China; 6 Department of Sport, Centre for Health and Exercise Science Research, Physical Education and Health, Hong Kong Baptist University, Kowloon Tong, 999077 Hong Kong; 7 School of Health and Life Sciences, Institute for Clinical Exercise & Health Science, University of the West of Scotland, South Lanark-shire, 999020, Glasgow, Scotland G72 0LH UK

**Keywords:** Badminton shoes, Questionnaire, Gender, Function, Foot injuries, Footwear

## Abstract

**Background:**

While the roles of injury prevention and performance enhancement have increasingly been investigated for badminton footwear, there is a lack of research on gender-specific badminton footwear. The purpose of this study was to examine the gender differences in footwear demands and foot injuries in badminton.

**Methods:**

The study was a cross-sectional survey, in which 326 recreational badminton players were recruited. The questionnaire was divided into four sections enquiring about the characteristics of (1) participant profiles, (2) importance of shoe properties (3) shoe complaints (4) and pain or discomfort in different foot regions. The Mann-Whitney U test and Wilcoxon Signed Ranks test were performed to determine the differences between genders and the differences between leg dominance, respectively. The significance level was set at 0.05.

**Results:**

Both males and females rated shoe fit as the most important features, followed by the overall comfort and injury protection. Females considered the shoe forefoot cushioning, comfort, breathability and colour as more important compared with the other properties, which showed distinct pattern differences from males. The shoe problem results indicated that plantar pain of the non-dominant foot was considered the most commonly reported footwear problem by both males and females. The problem of excessive arch-support on the dominant and non-dominant sides of male participants was significantly higher than females (*p* < 0.05). Occasional pain or frequent pain were mainly distributed in the forefoot, followed by the rearfoot and midfoot regions.

**Conclusion:**

There were small differences in footwear demand between the dominant and non-dominant sides, but several differences existed between females and males. The results from gender differences suggested that female shoes prefer a specific shoe last for better fit, rather than a modified version of male shoes. In the future, the design of badminton shoes should consider footwear demands and foot discomfort profiles in respective male and female badminton players.

## Introduction

Badminton is one of the most popular recreational sports worldwide. Biomechanical research of badminton sneakers typically focus on kinematic [1] and kinetic [2] variables associated with performances injuries in badminton. High-speed cameras and force platforms are frequently used to quantify movement characteristics and joint loading. However, these would cost enormous financial and human resources. In contrast, retrospective studies in hospital and clinics tend to underestimate the incidences and types of injuries [3], since injured amateur players often do not seek medical help, especially in the case of minor injuries (e.g. blisters, ankle sprain). Moreover, retrospective studies can employ personal interviews and structured questionnaires [4], which can allow researchers to gather a vast amount of data using reasonable human and financial resources. In addition to performance and injury perspectives, Llana et al. [5] raised the issue of the comfort of sport shoes. These fundamentals can be used in the design and development process of athletic shoes to improve shoe quality and specific function.

In a badminton competition, athletes intermittently perform repetitive strenuous movements including rapid acceleration, turning, sidestepping, cross-overstepping, lunging, jumping, high clear and smash, which exert high strains on the lower extremities, which may increase the risk of lower limb injuries [6]. Previous studies have shown that the modification of shoe constructions (e.g. midsole material, heel cup height, heel to toe drop) can induce a kinematic and kinetics adaptation, which influences sport performances and potential injury risks in various sports [7–9]. For instance, better shoe cushioning is related to better impact attenuation [1, 2, 10]; increased shoe bending stiffness is related to improve jumping, sprint and agility performances [2, 10]. Matching footwear requirements with movement characteristics can be beneficial to improve footwear development. Sport shoe characteristics for running, gym, football, basketball, and tennis have been previously studied using questionnaires [7, 11–14], but information for badminton has not been established. In addition, compared to males, a lower maximal stiffness and higher elasticity within the heel pad have been noted in females [15]. Furthermore, previous studies showed males have a significantly larger plantar fascia and heel fat pad thickness compared to females [16, 17]. Several investigations show that female feet were not just a scaled down version of male feet [18, 19] and female feet were characterized by a higher arch, shallower first toe, shorter length of the outside ball and smaller instep circumference. Other etiological factors including hip Q-angle, foot shape, body mass, muscle strength are different between genders [13], which results in distinct biomechanical alternations and thereby different footwear requirements between males and females [11]. Therefore, it can be assumed that badminton shoes need to be optimized with reference to these characteristics between genders in badminton. To date, there is a lack of research on badminton shoes based on gender-specific foot morphology.

The functional requirements of a shoe are multifaceted. While the foot is the only interface of the human body in contact with the ground, functional shoe constructions for good control, ground support, grip ability and agility are suggested to improve sports performance [20]. Inappropriate shoes and shoe fitting can cause several foot problems [21], such as blisters, squeezed toes, and soft tissue bruises [20]. The function of badminton shoes aims at minimizing the injury risks [22], whilst maximizing sports performance and comfort. Typically, badminton movements are highly asymmetrical with clear functional differences between the dominant and non-dominant legs. Hence, the purpose of this study was to investigate the shoes requirements, shoes problems/complaints and pain locations in males and females using supervised questionnaires. The results from this study can help to understand and collate badminton footwear requirements and foot pain mechanisms to provide insights of footwear feature recommendations and footwear development.

## Methods

### Study design and participants

This cross-sectional study was conducted at a recreational badminton match at Li-Ning Company (Beijing, China) in October 2019, with a total of 2000 participants. The basic inclusion criteria were: above 18 years old and had been regularly participating in badminton for the past 6 months. The exclusion criteria were: lower limb surgery or neurological injury. The supervised questionnaire contained the basic profile (height, weight, age and racket-hand/dominant leg), the importance of shoe properties, shoe complaints, and pain or discomfort across foot regions. Ethical approval was approved by the institutional Human Research Ethics Committee (IRB-2019-BM-0013) in accordance with the Declaration of Helsinki principles.

### Sample size

The sample size for this study was calculated using the online Sample Size Calculator (Raosoft Inc., Seattle, WA, USA, raosoft.com) with a 5% margin of error, 95% confidence interval, and 50% response distribution. A total of 500 recreational badminton players was approached while 326 returned their responds with their consent and participated in the study (response rate 65.2%).

### Data validity and collection

A total of 78 self-assessment items in the “importance of shoe properties”, “shoe complaints” and “pain or discomfort in different foot regions” sections of this study were assessed using the Likert scale, which showed a good reliability and validity to measure subjective perception [23, 24]. The reliability levels of the subscales were as follows: importance of shoe properties (Cronbach’s α = 0.94), shoe complaints (Cronbach’s α = 0.96), pain or discomfort across foot regions (Cronbach’s α = 0.63). Therefore, the reliability of the questionnaire in our study was acceptable. Bartlett spherical test and KMO (Kaiser-Meyer-Olkin) test were performed to ensure that the data characteristics were suitable for factor analysis. In the sample adequacy test, the KMO value of 0.812 is greater than 0.5, indicating that the questionnaire data was suitable for factor analysis. The Bartlett’s test result was X^2^ = 25,553.553, df = 3003, *P* = 0.000 < 0.05, confirming the validity of the questionnaire.

The questionnaire was completed by participants under the supervision of researchers, who provided guidance to ensure the validity of the data. In this study, the role of the researchers was to explain the definitions of the footwear and foot related terminology in order to avoid the misunderstanding of the technical terms, especially for the participants with little anatomy and/or footwear construction knowledge and to prevent the participants from random answers and missing answers, which greatly ensured the quality of the questionnaire.

The questionnaire was categorized into four sections: (1) participant profile, (2) importance of shoe properties, (3) shoe complaints, (4) pain or discomfort in different foot regions. All of the questionnaires were conducted when the participants were finished the competition.

In section one, participant profiles regarding gender, age, height, weight, racket-hand/ dominant leg were obtained. Section two and three required respondents to indicate subject’s rating on the importance of shoe properties and shoe complaints, respectively.

In section two, the importance of shoe properties was selected as the common shoe requirements during gameplays, which was established based on the previous studies on footwear properties in running, basketball and gym training [11, 12, 15, 25]. The assessed variables were overall evaluation of shoe, heel cushioning, forefoot cushioning, arch support, forefoot bending stiffness, traction/grip, durability, and stability. All respondents indicated their preferences on the 9-point Likert scale (1: extremely unimportant, 2: very unimportant, 3: unimportant, 4: somewhat unimportant, 5: neutral, 6: somewhat important, 7: important, 8: very important, 9: extremely important).

In section three, the footwear complaint was defined as any footwear problems encountered in badminton, including poor breathability, blisters, loose shoelaces, poor insole grip, forefoot squeezing toes (media-lateral), forefoot squeezing toes (dorsal), forefoot upper too hard, forefoot sole too hard (plantar pain), forefoot sole too soft (instability/sprain ankle), heel cup too soft (instability/sprain ankle), insufficient arch support, and excessive arch support. All of the shoe properties and footwear complaints were extracted from the previous studies on footwear comfort perception [9, 11, 12, 25] as well as advice from badminton coaches. All respondents gave their rating on the 9-point Likert scale (1: extremely comfortable, 2: very comfortable, 3: comfortable, 4: somewhat comfortable, 5: neutral, 6: somewhat uncomfortable, 7: uncomfortable, 8: very uncomfortable, 9: extremely uncomfortable).

**Fig. 1:**
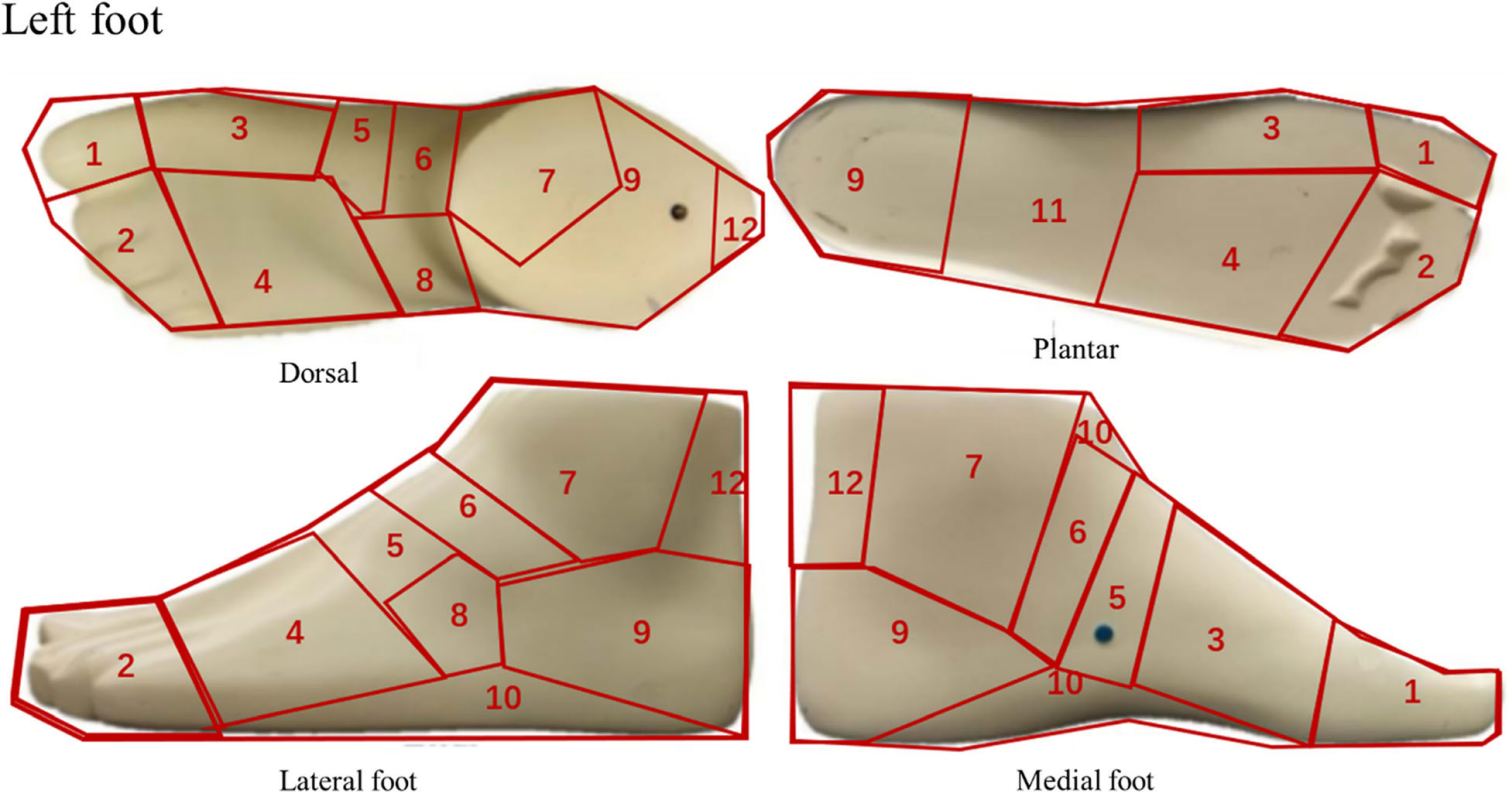
Diagram of the foot regions (Left foot). 1-Hallux, 2-Other four toes, 3-First MTP, 4-2nd-5th MTP, 5-cuneiform bone, 6-navicular bone, 7-Talus, 8-Cuboid bone, 9-Heel, 10-Soft tissues of the foot, 11-Arch, 12-Achilles’ tendon

In section four, respondents were asked to indicate any pain or discomfort at 12 ft regions (Fig. 1), including hallux, other four toes, first metatarsophalangeal (MTP), second-fifth MTP, cuneiform bone, cuboid bone, navicular bone, talus, heel, soft tissues of the foot, arch, and Achilles’ tendon, as described in previous studies [26, 27]. The degree of pain/discomfort was assessed by 3-point Likert scale (no pain, occasionally pain, and frequent pain) [28] for the dominant and non-dominant feet, respectively.

In addition, the subjective assessment was determined for respective dominant and non-dominant legs, as badminton is considered as a highly asymmetrical sport that results in uneven loading and movement characteristics. The sensitive dominant side was more suitable for athletes to use during competition, which may lead to the larger discrepancy of the strength and movement characteristics between dominant and non-dominant legs. Therefore, we also evaluated the requirements for footwear and pain on the dominant and the non-dominant sides [29].

### Data analysis

The data obtained were shown as means and standard deviations, as well as frequencies. The self-reported Likert scale was considered as non-parametric in nature. Moreover, additional Shapiro-Wilk tests showed that the data violated the normal distribution (*P* < 0.05). Therefore, the gender differences in all variables were analyzed using the Mann-Whitney U test, and the differences between the dominant and non-dominant feet were analyzed using the Wilcoxon Signed Ranks Test. The significance level was set at *P* < 0.05. All statistical analyses were conducted using SPSS 21.0 (SPSS Inc., Chicago, IL, USA).

## Result

### Characteristics of the participants

Altogether 326 recreational badminton players (200 males, 126 females, all Chinese citizens) participated in the experiment. Their mean age were 30.9 ± 11.8 years and 33.18 ± 12.1 years, respectively. The body mass index of males was 23.3 ± 3.4 and 21.3 ± 2.7 for females, respectively. The participants were randomly recruited from the badminton tournament, which was held over a month.

### Importance of shoe properties

In Table 1, both males and females rated shoe fit as the most important variable, followed by shoe comfort and injury protection. The Mann-Whitney U test showed significant differences in the importance of some shoe features between males and females. Females reported higher importance of forefoot cushioning, comfort, breathability, colour and upper durability than males (*P* = 0.002, 0.032, 0.043, 0.049 < 0.05).

**Table 1:**
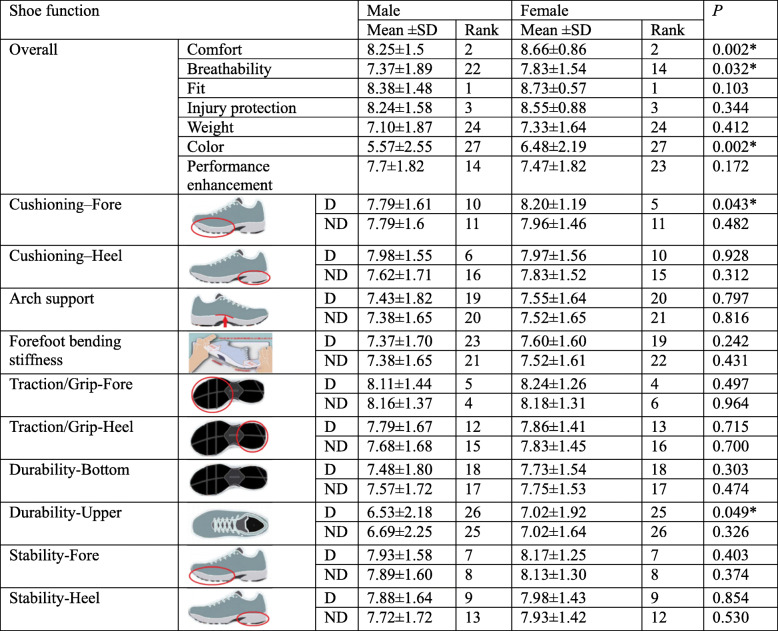
Importance of shoe properties between genders

*D* Dominant, *ND* Non-dominant. *Indicates a significant difference, *P* < 0.05

Wilcoxon Signed Ranks Test was used to compare the importance of shoe characteristics between dominant and non-dominant sides, respectively (Table 2). For males, heel cushioning and heel stability were more important (*P* = 0.000, 0.010), while the upper durability were less important on the dominant side (*P* = 0.002) compared with the non-dominant side. For females, forefoot cushioning on the dominant shoe was significantly more important than the non-dominant shoe (*P* = 0.019).

**Table 2:**
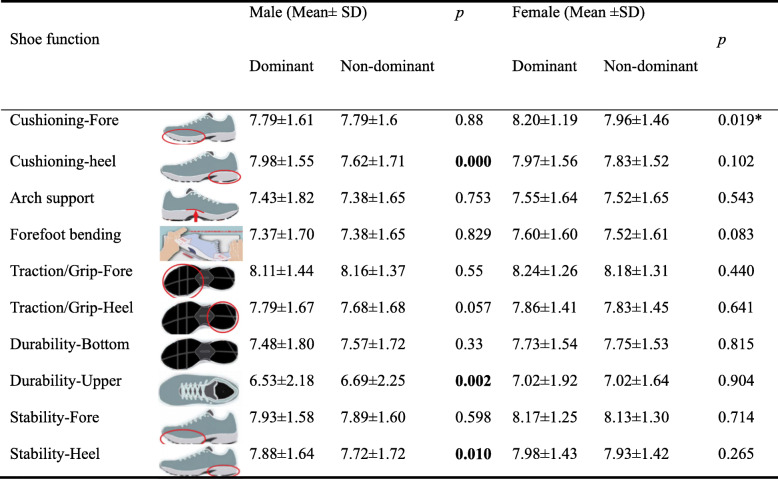
Importance of shoe properties between dominant and non-dominant sides

*Indicates a significant difference, *P* < 0.05

### Shoe problems/complaints

Descriptive statistics showed that none of the shoe problems were extremely serious, however individual differences were large (Table 3). By ranking the severity of shoe problems, plantar pain attributed to “sole too hard” of non-dominant foot was considered as the most serious footwear problem by both males and females. In addition, for males, the second most crucial factor was also the plantar pain attributed to “sole too hard” of the dominant foot. For females, the next shoe problem ranking was squeezing toes (medial- lateral), forefoot upper, and sole too hard on the dominant foot (Table 3).

**Table 3:**
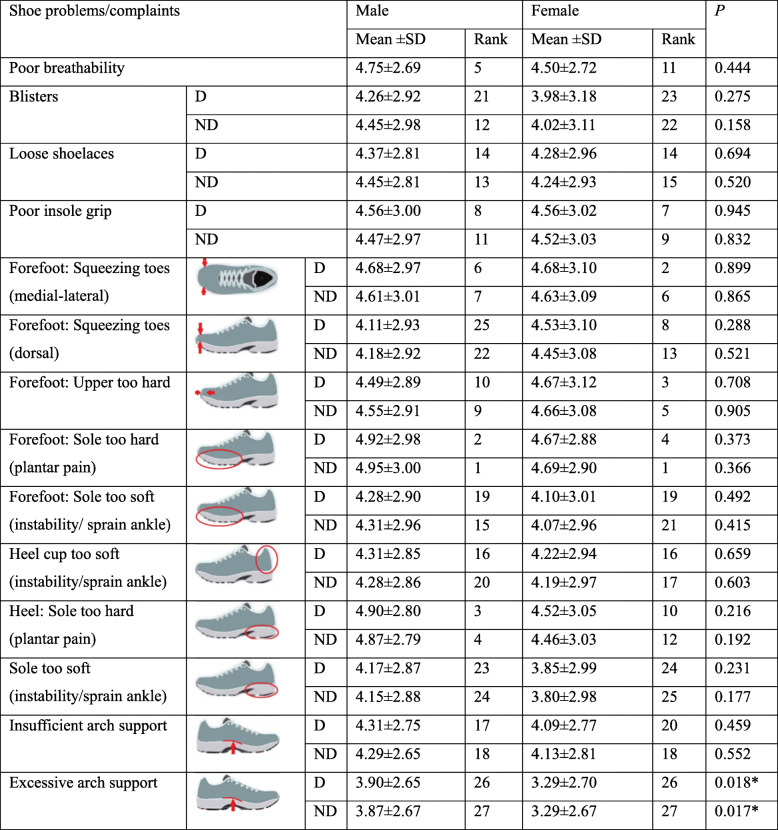
Shoe problems/complaints between genders

*D* Dominant, *ND* Non-dominant. *Indicates a significant difference, *P* < 0.05

The Mann-Whitney U test reported that the shoe problem of excessive arch support on both dominant and non-dominant sides were significantly higher in males than females (*P* = 0.017, 0.018, Table 3). Wilcoxon Signed Ranks test showed no significant difference between dominant and non-dominant sides (Table 4).

**Table 4:**
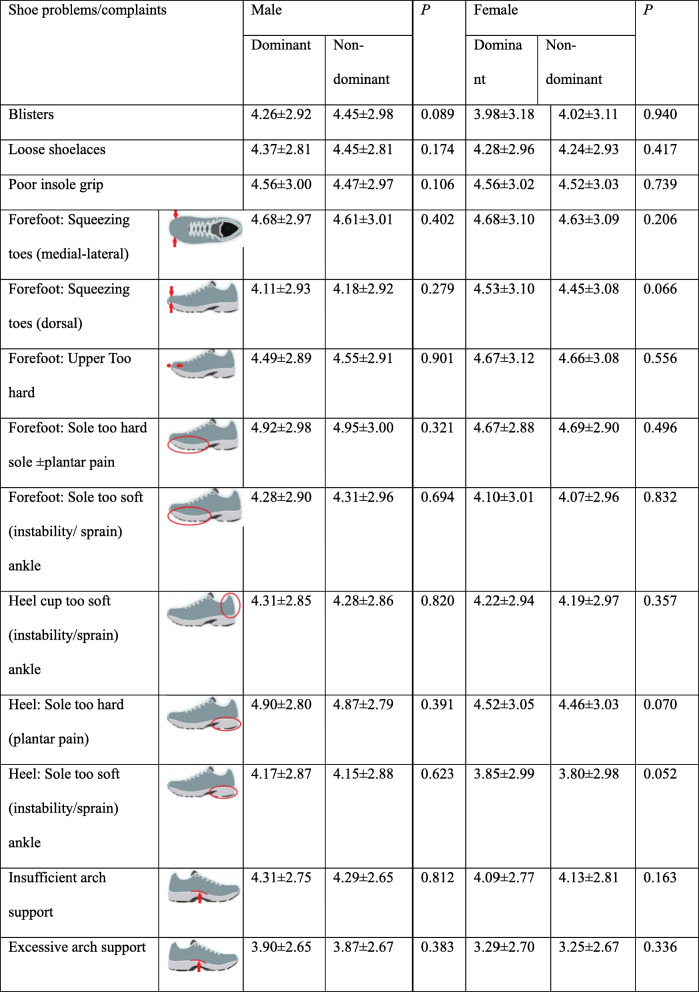
Shoe problems/complaints between dominant and non-dominant sides

### Pain or discomfort in different foot regions

The foot regions with occasional pain or frequent pain were distributed in the forefoot, followed by rearfoot and midfoot regions (Table 5). The gender difference results showed that occasional pain in the hallux on both dominant and non-dominant feet was more likely in females than males (*P* = 0.017, 0.032). On the other hand, the heel frequent pain on the dominant and non-dominant sides of males were significantly higher than that of females (*P* = 0.009, 0.023). Similarly, the soft tissue of the foot on the dominant side was significantly higher in males than females (*P* = 0.028).

**Table 5:** Foot pain/ discomfort locations between genders

Foot regions		NP (%)		OP (%)		FP (%)		*P-* values
		male	female	male	female	male	female	Male vs. female
Hallux	D	87.5	77	10	21.4	2.5	1.6	0.017*
	ND	89	80.2	8	16.7	3	3.2	0.032*
Other toes	D	93	87.3	6	11.9	1	0.8	0.088
	ND	94	93.7	4.5	6.3	1.5	0	0.926
1st MTP	D	76.5	76.2	21	20.6	2.5	3.2	0.747
	ND	74.5	78.6	22.5	19	3	2.4	0.669
2nd-5th MTP	D	84.5	81.7	14	15.1	1.5	3.2	0.484
	ND	89	87.3	10.5	11.9	0.5	0.8	0.638
Cuneiform bone	D	97.5	95.2	2.5	2.4	0	2.4	0.259
	ND	96	95.2	2.5	4.8	0.5	0	0.418
Navicular bone	D	98.5	99.2	1.5	0	0	0.8	0.580
	ND	99.5	97.6	0.5	2.4	0	0	0.569
Talus	D	95.5	90.5	4.5	8.7	0	0.8	0.070
	ND	96.5	97.6	3.5	2.4	0	0	0.950
Cuboid bone	D	100	99.2	0	0.8	0	0	0.208
	ND	97.5	97.6	2	1.6	0.5	0.8	0.950
Heel	D	70.5	84.1	23	9.5	6.5	6.3	0.009*
	ND	77.5	88.1	16.5	6.3	6	5.6	0.023*
Soft tissues	D	93	98.4	6.5	1.6	0.5	0	0.028*
	ND	95	96.8	4.5	2.4	0.5	0.8	0.435
Arch	D	96.5	96	3.5	2.4	0	1.6	0.807
	ND	96	95.2	3	4	1	0.8	0.747
Achilles’ tendon	D	99	94.8	1	1.6	0	0	0.640
	ND	99.5	100	0.5	0	0	0	0.427

*D* Dominant, *ND* Non-dominant, *NP* No pain, *OP* Occasional pain, *FP* Frequent pain; *indicates a significant difference, *P* < 0.05

## Discussion

Badminton requires athletes to perform substantial explosive movements on joint loading [29, 30], which could be related to various extremely rapid and intense activities during the game [31]. The foot is susceptible to considerable high amount of pressure, which increases the risks of potential foot injuries [32]. Badminton shoes are clearly different from other sports shoes, and they must be functionally suitable for the characteristics of badminton players [1]. The basic requirements of badminton footwear usually focus on the soles, the weight and appearance [8, 29]. It is generally believed that the correct shoe shape is obtained by matching shoe shape to foot shape [33]. Therefore, considerations of the gender differences in foot shape design is essential to the proper design of both male and female footwear [18]. However, it is still questionable if male and female athletes would demonstrate different footwear requirements, foot complaints and foot injury locations, since there are considerable anthropometrical and biomechanical differences between genders. The objective of this cross-sectional survey was to investigate the shoe requirements, shoe problems/complaints and pain locations in males and females using supervised questionnaires. As a non-contact sport, badminton has obvious laterality in its lower limbs. Badminton involves repeated rapid forward lunges, the dominant leg bears a greater load than the non-dominant leg. Therefore, the dominant and non-dominant side characteristics of badminton shoes should also be examined [34]. The results from this study can provide insights for badminton footwear development.

Our results showed that the fit and comfort of badminton shoes were recognized as the most important shoe feature in both males and females. This is similar to previous research on running, soccer, gym, basketball and tennis footwear, which also reported fit and comfort as the most important shoe features [1, 11–14]. Moreover, another research studying shoe comfort during standing tasks, preferred footwear conditions were shown to result in the lowest levels of lower extremity and back pain. In addition to injuries, it has been suggested that footwear comfort is related to sport performances [35, 36]. Some studies have found significant improvements in running economy when wearing their most comfortable/preferred shoe conditions [37].

Shoe fit is a prerequisite to shoe comfort as well as sports performance, fatigue and injury prevention [14, 38, 39]. Comfortable fit is also considered essential for shoe performance [40–42]. Fit and comfort are closely related to shoe design [43]. Although shoe fit and comfort were ranked as important by both genders, the higher importance of fit and comfort was found in female players. One possible explanation is that females may have different foot shape, with wider forefoot and narrower heel, compared with males [18, 19, 44]. However, most female sports shoes are scaled down versions of male shoes [44], resulting in potential concerns on shoe fit. Another explanation is due to the higher hallux valgus angles found in females than males [45–50], which would result in more sensitivity to shoe upper pressures exerted on the hallux and therefore higher frequency of discomfort of the female hallux.

In our study, females reported importance for shoe color, dominant forefoot cushioning and upper durability than the males, suggesting that colour should be always considered in female footwear. Biomechanically, the function of shoes is minimally affected by color. From the cognitive science perspective, colour can influence human cognition, perception and behaviour, which may in turn has great impact on motor performances [6, 51–53]. The earliest study investigating the color of badminton shoes [54] indicated that badminton shoes should concentrate on exciting colors (e.g. red) and material combinations, which could help to improve the wearer’s sports performance perception.

Compared to males, females have wider pelvis width, which is associated with greater genu valgus, greater external tibial torsion and a greater Q-angle. Previous work has shown that female athletes have higher knee injury rates than male athletes in many court sports such as basketball and soccer [55], which is partly consistent with our survey results. Our female respondents rated shoe cushioning as one of the important shoe features in badminton and the need for shoe cushioning was more important in the dominant leg compared with the non-dominant leg to lower the impact of the lower limbs during exercises. Since females have narrower heel and higher medial arch than males [19], females prefer shoes with better upper fit and durability.

Based on our shoe problem/complaints findings, there were no gender differences found for most of shoe problems/complaints in regular sports. Due to the different anatomical structures of male and female feet, female arches are higher than males. Excessive arch support causes excessive ankle varus, which is suggested to increase the risk of ankle sprain [56, 57]. Subjectively, athletes exhibit differences in perceived shoe stiffness based on mechanical properties. As a result, soft soles were more popular than hard soles, and shoes with a stiffer forefoot were considered particularly uncomfortable for recreational athletes [58]. Our foot discomfort and pain results showed that the plantar region was the most susceptible to discomfort or pain regardless of gender. Together with the findings from the “importance of shoe properties” section, which showed a higher demand on fore-foot cushioning. Moreover, our recreational badminton athletes complained of hard forefoot soles. Wearing shoes may alter cutaneous proprioception, mainly due to mechanoreceptors on the plantar surface [59, 60]. The cutaneous proprioception is one of the most important sensory systems to regulate the postural stability [61]. Furthermore, ankle proprioception is a key part of the feedback loop that is regulated by the central nervous system to maintain a stable upright posture while standing quietly. In a similar vein, badminton shoes might affect this proprioceptive process by changing the structure of the shoe, which could alter the sensory inputs on the foot and thus influence postural strategy [62]. In the future, forefoot cushioning should be improved together with the individual perception to minimize the potential risk of foot and lower-limb injuries.

Several limitations need to be considered when interpreting our data. First, badminton athletes did not wear the same shoes, which may result in different wearing experiences and footwear preferences. Second, only recreational athletes and adult athletes were recruited for the study. Our results may not be generalizable to athletes at elite or lower playing levels. Highly skilled athletes demonstrated larger lunge distance and landing angles as well as higher movement intensities, implying that different shoe demands and foot pain/injury profiles.

## Conclusion

This study provided comprehensive information related to badminton shoe demands, shoe problems/complaints, and discomfort locations in respective leg-dominance and genders. Good fit and comfort are considered as the most important shoe features for badminton shoes. The differences of shoe problems or complaints between dominant and non-dominant shoes were not obvious, while there were clear differences in shoe feature demand between females and males. These findings suggest that female-specific shoes are recommended for better shoe fit and comfort, as indicated by the anthropometrical differences between genders.

## Data Availability

The datasets used and/or analysed during the current study are available from the corresponding author on reasonable request.
